# Supplementary material for: Onset of persistent surface ocean oxygenation during the Great Oxidation Event
Source: Nat Commun. 2025 Dec 9;16:10190. doi: 10.1038/s41467-025-66323-5 (PMC12690087; doi:10.1038/s41467-025-66323-5)
Supplement: Supplementary file 1 — Supplementary Information [file 41467_2025_66323_MOESM1_ESM.pdf]

## Supplementary Text

Reconciling vanadium isotope constraints on marine oxygenation with possible reappearances of S-MIF

Sporadic, short returns of S-MIF in the upper Timeball Hill Formation, potentially suggesting short-lived, atmospheric deoxygenation events between ~2.32 and 2.22 Ga[1], are mostly observed in samples deemed unsuitable for V isotopic analyses due to oxic local redox conditions (Fig. 1). The origin of these younger returns of non-zero S-MIF are still heavily debated[1–3], and due to the unresolved nature of this debate and its peripheral connection to the data presented herein, they are only briefly discussed in connection to V isotopic data below. The $\delta^{51}V_{auth}$ dataset does include two isolated samples with non-zero S-MIF, at 1091 and 943 m depth. In each case, $\delta^{51}V_{auth}$ in these samples are indistinguishable from the upper Timeball Hill Formation samples lacking S-MIF (Fig. 1G), suggesting that $pO_2$ perturbations at this time were not transferred to the oceans in a manner that impacted the marine V cycle. Two phenomena that may each fully or partially explain these observations concern the duration of any possible short-lived S-MIF returns, and the partitioning of V between different, seafloor redox sinks. Statistical approaches were recently used to show that the development of short S-MIF reappearances in the Timeball Hill Formation sections[1,2] requires that any ephemeral returns to an anoxic atmospheric redox state would have lasted for as little as tens of thousands of years[3]. These timescales overlap with the modern (91 kyr) V residence time in seawater. Even accounting for a shorter V residence time under more anoxic ocean conditions associated with a smaller seawater V reservoir, it is unclear that such a short-lived perturbation in surface marine $O_2$ forcing would have propagated its effect to the global $\delta^{51}V_{sw}$ value. This is because global ocean geochemical responses to forcings are expected to be best expressed when forcings are longer in duration than the residence time of the element of interest[4].

## Supplementary Figures

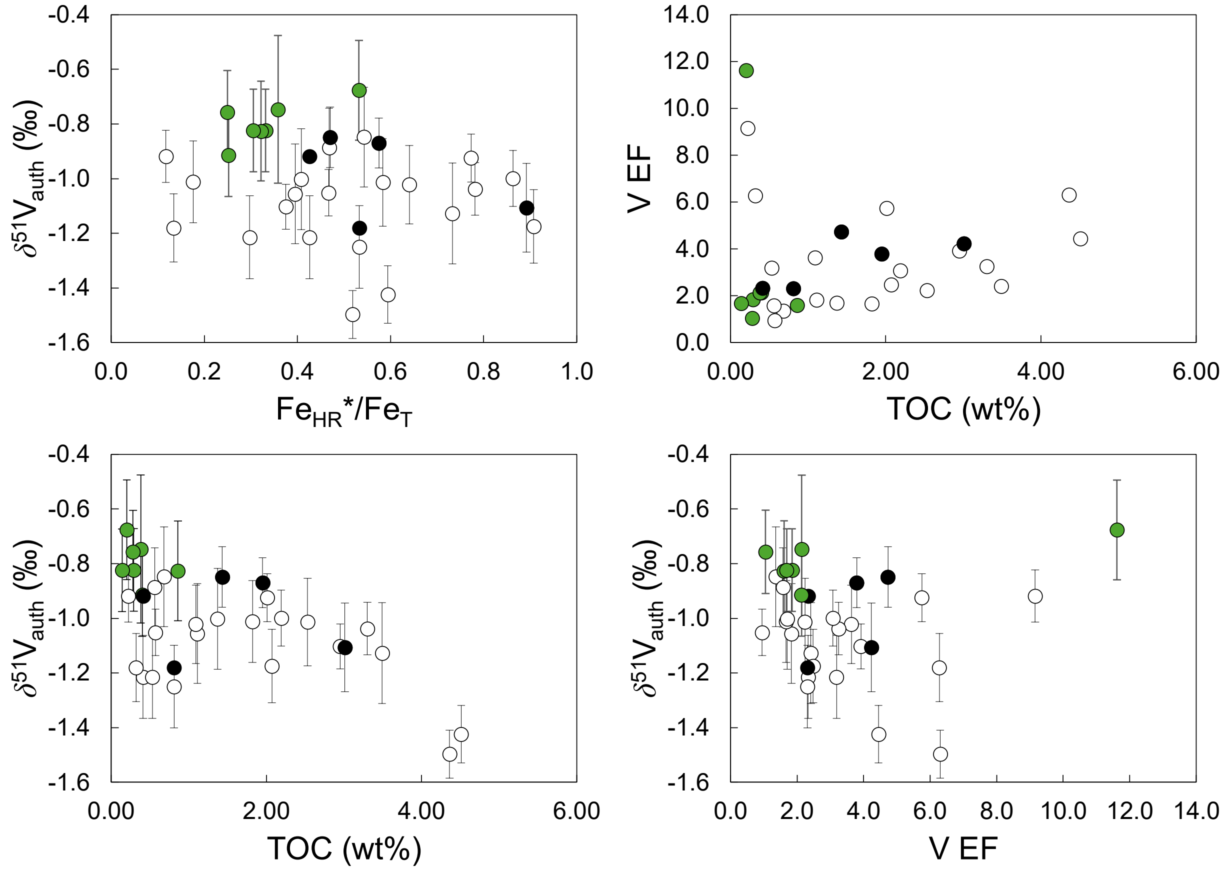


**Fig. S1: Crossplots of $\delta^{51}V_{auth}$ and local redox indicators for the Rooihoogte and Timeball Hill formations.** Lower section is shown in white symbols with euxinic lower section samples picked out with black symbols; upper section shown in orange. Lack of systematic covariation between $\delta^{51}V_{auth}$ and $Fe_{HR}*/Fe_T$, TOC, V EF suggests that isotopic fractionation between seawater and authigenic V was not systematically controlled by local redox conditions. A rough negative covariation of some lower section $\delta^{51}V_{auth}$ and V EF data may resemble the signatures of local seawater V drawdown seen in other anoxic Precambrian sediments[5], but a relationship is not well developed enough to allow more precise seawater $\delta^{51}V$ reconstruction than the qualitative ranges applied in the main text. Lower V EF in the upper than lower section appears to be related to limited TOC with which to deliver authigenic vanadyl to sediment, rather than local drawdown of the seawater V reservoir. Error bars for $\delta^{51}V_{auth}$ are 2 SD of reproducibility on either the individual sample or the BDH chemicals V solution standard, whichever is larger.

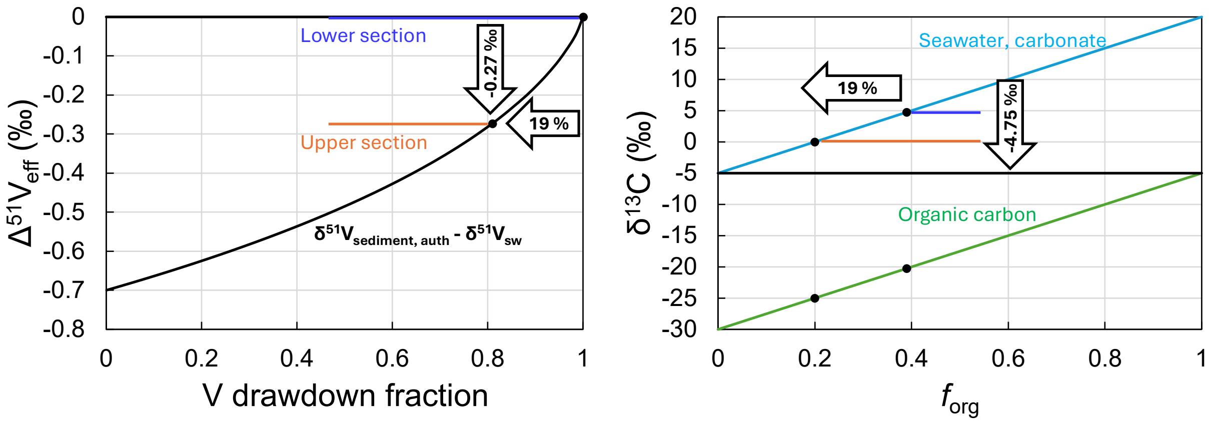


**Fig. S2: Simplified isotope fractionation models for organic carbon burial-driven mass balance of V and C isotopes.** Left: Rayleigh distillation model showing the effective offset $\Delta^{51}V_{eff}$ between seawater and the cumulative sedimentary V pool drawn down by adsorption of vanadyl to organic matter with an instantaneous isotopic difference of -0.7 ‰. The smallest possible fractional change in V drawdown to explain a -0.27 ‰ decrease in $\Delta^{51}V_{eff}$ inferred to drive the shift in reconstructed seawater $\delta^{51}V$ going from the lower to upper sections is 19 %, when going from 100% to 81% V drawdown as vanadyl sorbed to organic carbon. Right: Simple 2-component C isotope mass balance for the oceans assuming that C is added to oceans with $\delta^{13}C$ = -5 ‰ and drawn down to an unfractionated carbonate sink and an organic carbon sink offset by -25 ‰ from seawater. A proportionate 19 % decrease in organic carbon burial would drive a 4.75 ‰ decrease in marine DIC and carbonate $\delta^{13}C$ that is not observed in the rock record.

**Supplementary Data S1. (separate file)**

Excel file containing geochemical data including V isotope data for shale samples from the EBA-2 drill core, Rooihoogte and Timeball Hill formations, South Africa.

## References


1. Poulton, S. W. *et al.* A 200-million-year delay in permanent atmospheric oxygenation. *Nature* 1–5 (2021) doi:10.1038/s41586-021-03393-7.
2. Izon, G. *et al.* Bulk and grain-scale minor sulfur isotope data reveal complexities in the dynamics of Earth's oxygenation. *Proc. Natl. Acad. Sci.* **119**, e2025606119 (2022).
3. Uveges, B. T., Izon, G., Ono, S., Beukes, N. J. & Summons, R. E. Reconciling discrepant minor sulfur isotope records of the Great Oxidation Event. *Nat. Commun.* **14**, 279 (2023).
4. Richter, F. M. & Turekian, K. K. Simple models for the geochemical response of the ocean to climatic and tectonic forcing. *Earth Planet. Sci. Lett.* **119**, 121–131 (1993).
5. Fan, H., Ostrander, C. M., Auro, M., Wen, H. & Nielsen, S. G. Vanadium isotope evidence for expansive ocean euxinia during the appearance of early Ediacara biota. *Earth Planet. Sci. Lett.* **567**, 117007 (2021).
