# Supplementary material for: Onset of persistent surface ocean oxygenation during the Great Oxidation Event
Source: Nat Commun. 2025 Dec 9;16:10190. doi: 10.1038/s41467-025-66323-5 (PMC12690087; doi:10.1038/s41467-025-66323-5)
Supplement: Supplementary file 2 — Description of Additional Supplementary Files [file 41467_2025_66323_MOESM2_ESM.pdf]

Description of Additional Supplementary Files

Supplementary Movie 1: Geochemical data including V isotope data for shale samples from the EBA-2 drill core, Rooihoogte and Timeball Hill formations, South Africa.
